# Complete mitochondrial genome of the *Minois paupera* Alphéraky, 1888 (nymphalidae: satyrinae) and its phylogenetic analysis

**DOI:** 10.1080/23802359.2024.2361704

**Published:** 2024-06-13

**Authors:** Wenqian Hu, Kangshan Mao, Liang Dou

**Affiliations:** College of Life Sciences, Key Laboratory of Bio-Resources and Eco-Environment of Ministry of Education, Key Laboratory of Conservation Biology on Endangered Wildlife of Sichuan Province, Sichuan University, Chengdu, China

**Keywords:** *Satyrinae*, *Minois paupera*, mitochondrial genome, phylogeny

## Abstract

The present study firstly reported a complete mitochondrial genome of *Minois paupera* (Alphéraky, 1888), a Satyrinae species endemic to China. This mitogenome is circular, 15,213 bp in length, and consists of 37 typical mitochondrial genes, including 13 protein-coding genes (PCGs), 22 tRNAs, and two rRNAs. The phylogenetic position was inferred using 31 previously published complete mitogenomes, and the results reveal that *M. paupera* is the most closely related to *Minois dryas*. The complete mitogenome of *M. paupera* provides useful genetic information for further research on the phylogeography and phylogeny of the genus *Minois.*

## 1. Introduction

The genus *Minois* Hübner, which belongs to ‘Satyrinae,’ known as the mimicry eye spots, is mostly distributed in Palearctic, including one widespread species and several regional endemic species. The genetic relationships of species under this genus are still unclear (Sbordoni et al. 2018). In addition, the unique geographical distribution pattern of this population may be related to historical geological events, which can be used as an excellent research material for biogeography and species differentiation. However, up to now, there are only one complete mitochondrial genome of the dispersed species *Minois* had been reported, hindering further studies of phylogenetic and phylogeography.

*Minois paupera* (Alphéraky, 1888), belongs to *Minois* and is endemic to China (including in Gansu, Qinghai, Sichuan, N.W. Yunnan, S.E. Tibet) (Lang 2022). The previous studies mainly focused on taxonomy and mostly based on morphological features, lacking the support of molecular evidence. In this study, we sequenced the complete mitochondrial DNA genome of *M. paupera* to provide baseline data for better understanding its relationship within the genus *Minois*.

## 2. Materials and methods

### 2.1. Sample collection and preservation

**Figure 1.**
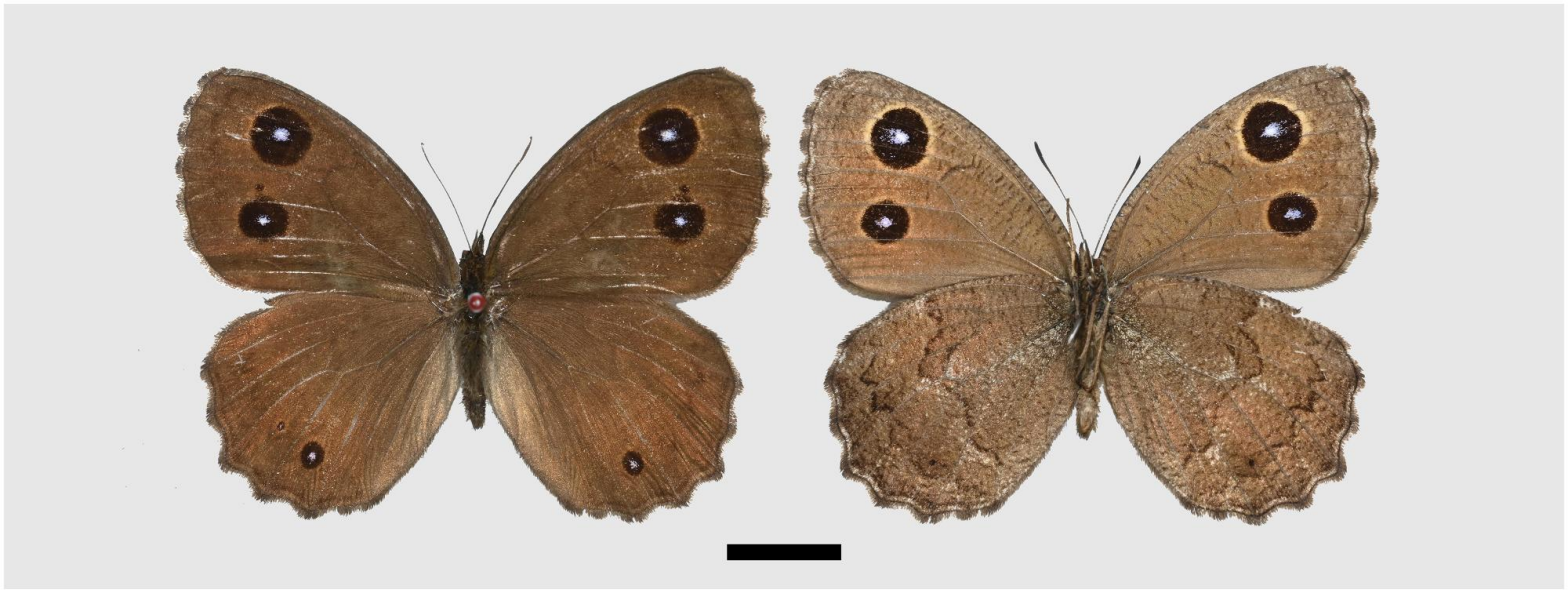
The specimen of *Minois paupera* (Alphéraky, 1888) used in this study, upperside on the left, underside on the right, scale bar = 10 mm. Photographed and processed by wen-qian Hu.

The specimen was collected from Yongjing County, Gansu Province (103°23'32.65"E, 36°4'5.50"N, 2,160 m). Three legs on the same side were extracted and preserved in ethanol. In terms of morphological characteristics, the dorsal surface yellowish or orange rings around the postdiscal ocelli present on both wings. In addition, male dorsal forewing brand well developed in spaces 1b and 2, and often extending into space 3. The dorsal hindwing postdiscal ocellus in space 2 well developed (Lang 2022). The spread male specimens (Figure 1) were deposited in the Natural History Museum of Sichuan University, Chengdu, China (specimen numbers: SZM742205, contact person: Liang Dou, douliang@scu.edu.cn).

### 2.2. DNA extraction sequencing and genomic assembling

Genomic DNA was extracted from the three legs of a single individual butterfly using the Sangon Rapid Animal Genome DNA Isolation Kit (Shanghai, China). The library preparation and next generation sequencing was finished by Sangon Biotech (Shanghai) Co., Ltd. The libraries were pooled and loaded on Novaseq 6000 (Illumina, San Diego, USA) sequencer by 2 × 150bp paired end sequence kit according to the manufacture’s instructions.

Rawbases yielded at least 6 GB were used for downstream analysis. All of the raw reads were trimmed by Fastqc 0.11.2 and assembled the raw sequence reads into contigs by SPAdes 3.15 (Bankevich et al. 2012). The coverage depth is 1x∼500x, mean: 190x (Figure S1, Table S1). Finally, complete mitochondrial genome was achieved using the contigs hit against the reference mitochondrial genome as seed sequence through the software MITObim 1.9.1 (Hahn et al. 2013).

### 2.3. Annotation and phylogenetic analysis

The CDS gene boundary was obtained by reverse comparison with the reference genomes of closely related species through NCBI Blast+ 2.28 and GeneWise (Birney et al. 2004). MiTFi (Jühling et al. 2012) was used to obtain tRNA sequence annotation, cmsearch (rfam.cm) identifies non-coding RNA by comparison. Finally, summarizes and collates the complete annotation results. The phylogenetic tree was constructed using PhyloSuite 1.2.3 (Zhang et al. 2020) based on concatenated nucleotide sequences of 13 PCGs and two rRNAs of *M. paupera*, other 29 representatives from 3 subfamilies of the Nymphalidae, and two outgroup species from Lycaenidae. The nucleotide sequences datasets were initially aligned in batches with MAFFT v7.505 (Katoh and Standley 2013). Ambiguously aligned fragments within the alignments of the 13 PCGs and gap sites within the rRNA sequences were subsequently removed using Gblocks 0.91b (Talavera and Castresana 2007) and trimAl v1.2rev57 (Capella-Gutiérrez et al. 2009), respectively. Maximum likelihood (ML) phylogenies were inferred using IQ-TREE v2.2.0 (Nguyen et al. 2015) under the optimal GTR + F + R5 model identified by ModelFinder (Kalyaanamoorthy et al. 2017) for 5000 ultrafast (Minh et al. 2013) bootstraps. In addition, CodonW (http://codonw.sourceforge.net//culong.html) was used to analyze The Relative Synonymous Codon Usage (RSCU).

## 3. Results and discussion

### 3.1. Characteristics of M. paupera genome

**Figure 2.**
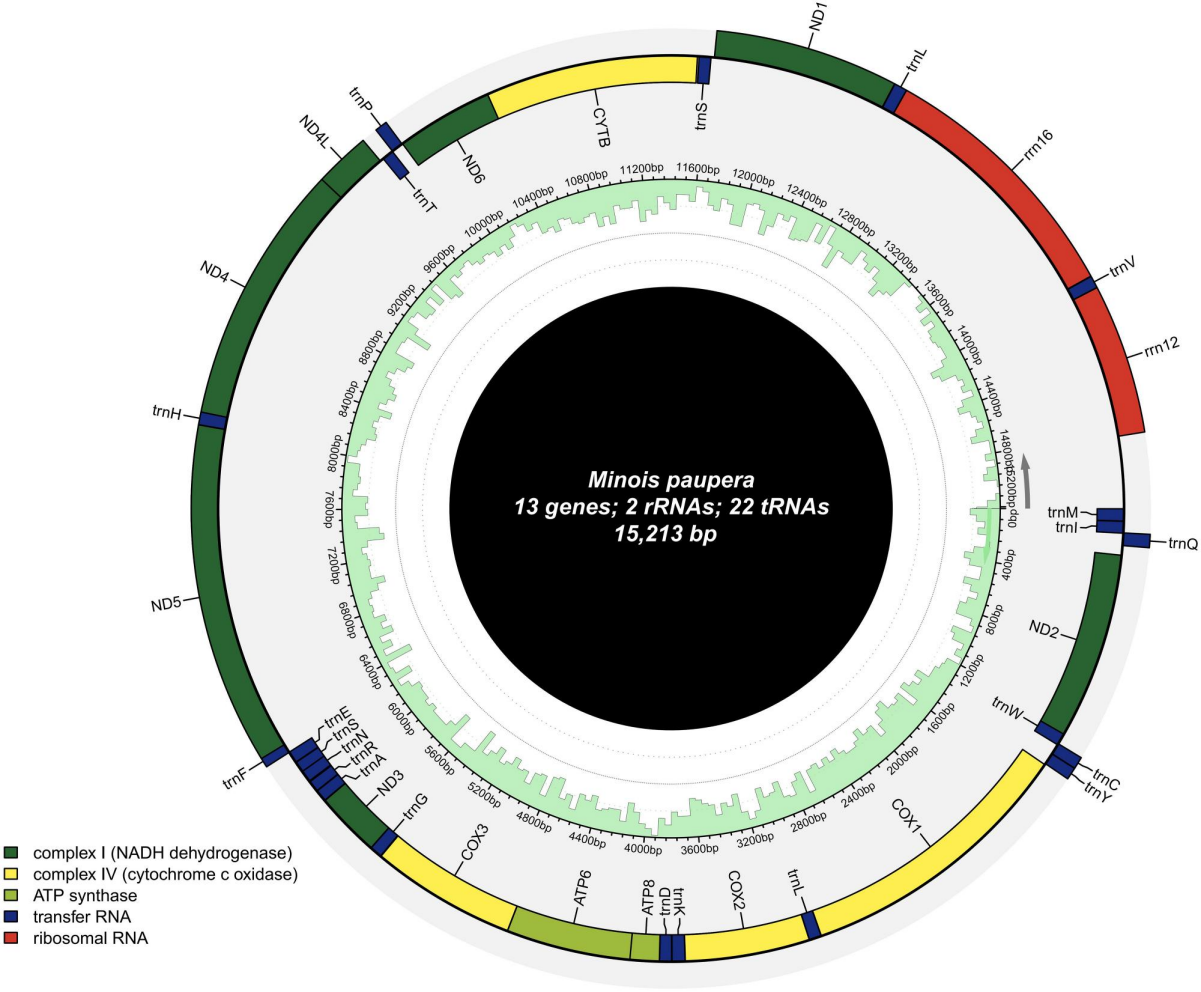
The circular-mapping mitochondrial genome of *Minois paupera*: the total length was 15,213 bp, which was divided into 37 genes, including 13 PCGs, 22 tRNAs, two rRNAs.

The mitochondrial genome of *M. paupera* is 15,213 bp in length (GenBank accession number: OR944656), with AT content (80.2%) significantly higher than GC Ratio (19.81%). The genome contains 13 protein-coding genes (PCGs), 22 transfer RNA genes (tRNAs), and two ribosomal RNA genes (rRNAs), The plus (+) strand encodes *nad2*, *cox1*, *cox2*, *atp8*, *atp6*, *cox3*, *nad3*, *nad6*, and *cytb*, while the minus (−) strand encodes *nad1*, *nad4l*, *nad4* and *nad5* (Figure S2). The gene content and arrangement are highly conserved, exhibiting typical characteristics of other *Minois* species (Yang et al. 2020) (Figure S2, Table S2). In addition, codon bias analysis reveals that among the amino acids, arginine, leucine, and serine exhibit the highest codon bias scores, indicating the most pronounced codon preference. These amino acids show the highest level of genetic variation within the genome (Figure S3) (Figure 2).

### 3.2. Phylogenetic position

**Figure 3.**
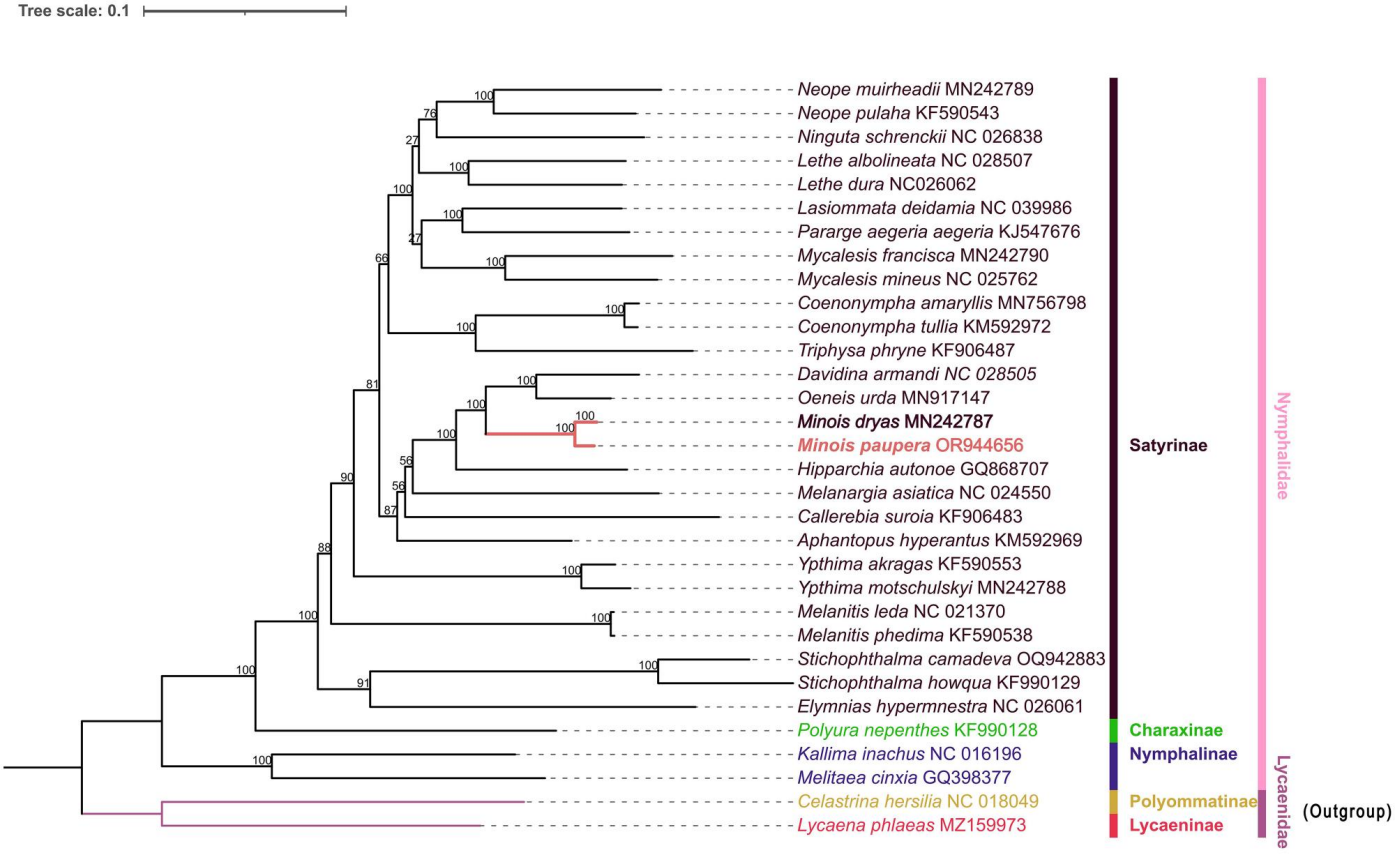
The Maximum likelihood (ML) phylogenetic tree contains *Minois paupera* (Alphéraky, 1888) (marked with bold font), 29 nymphalidae and 2 lycaenidae, The numbers on each branch are bootstrap support values out of 5000 replicates. The GenBank accession number followed by each species name, and the vertical lines on the side indicate families and subfamilies. The following sequences were used: MN242789 (Yang et al. 2020), KF590543 (Wu et al. 2014), NC026838 (Fan et al. 2016), KJ547676 (Teixeira da Costa 2016), MN242790 (Yang et al. 2020), NC025762 (Tang et al. 2014), MN756798 (Zhou et al. 2020), KF906487 (Zhang et al. 2016), MN917147 (Zhou et al. 2020), MN242787 (Yang et al. 2020), GQ868707 (Kim et al. 2010), NC024550 (Huang et al. 2016), KF590553 (Wu et al. 2014), MN242788 (Yang et al. 2020), NC021370 (Shi et al. 2013), KF590538 (Wu et al. 2014), NC016196 (Qin et al. 2012).

The ML phylogenetic tree reveals that *M. paupera* is most closely related to *M. dryas*, while the genus *Minois* forms a cluster with *Oeneis* and *Davidina*, both supported by 100% values. Furthermore, the monophyly of the genus *Minois* is well supported in phylogenetic analyses, which aligns with previous phylogenetic studies (Yang et al. 2020; Zhou et al. 2020) and morphological classification research (medium or large size butterflies; antennal club slender, not spatulate as in genus *Hipparchia*; forewing subcostal vein dilated at base, vein 1b not thickened; hindwing outer margin scalloped, slightly in males, more deeply in females; male upf androconial patch present or absent; forewing two large single-pupilled postdiscal ocelli always present in space 2 andspace 5) (Lang 2022). Notably, *Minois* species have traditionally been classified within the genus *Satyrus*, however, there is a dearth of comprehensive molecular data for further validation. Moreover, except for *M. dryas*, all species in this genus are exclusively found in China and exhibit predominantly regional distributions, but the genetic relationship between these species is still unclear. Therefore, it is imperative to conduct further molecular investigations at the mitochondrial genome level or beyond to elucidate the phylogenetic relationships within this genus (Figure 3).

## 4. Conclusions

In the present study, the complete mitogenome of *M. paupera* was assembled and analyzed. It will provide useful information for improving the taxonomic system of *Minois*. We found the phylogenetic position of *M. paupera* within the subfamily of Satyrinae was determined, and the results showed that *M. paupera* was closely related to *M. dryas*, and the gene content and arrangement of the newly sequenced mitogenome are similar to those of other determined mitogenomes of Satyrinae. It also provides baseline data for further molecular verification of the biogeographical evolution and phylogenetic relationships within the Satyrinae.

## Data Availability

The genome sequence data that support the findings of this study are openly available in GenBank of NCBI at (https://www.ncbi.nlm.nih.gov/nuccore/OR944656) under the accession no. OR944656. The associated BioProject, SRA, and Bio-Sample numbers are PRJNA1053844, SRR27238771, and SAMN38873769 respectively.
